# Whole-cell-catalyzed cinnamoylation of phillyrin in natural deep eutectic solvent-based co-solvent systems

**DOI:** 10.3389/fmicb.2026.1906247

**Published:** 2026-08-21

**Authors:** Rongling Yang, Ziling Huang, Yuli Tong, Dong Xu, Xiaoyan Li, Yanjin Li, Chun Zhu, Xiangjie Zhao

**Affiliations:** School of Life Science and Food Engineering, Huai'an University, Huai'an, Jiangsu, China

**Keywords:** *Aspergillus oryzae*, cinnamoylation, natural deep eutectic solvents, phillyrin, whole-cell biocatalysts

## Abstract

Phillyrin is a critical bioactive constituent of *Forsythiae Fructus*, exhibiting multiple pharmacological properties, but suffers from poor membrane permeability and low oral bioavailability. In this study, we developed a novel, green method employing whole-cell biocatalysts and natural deep eutectic solvents (NADESs) for the cinnamoylation of phillyrin to improve its liposolubility and bioactivity. *Aspergillus oryzae* whole cells showed high catalytic activity and stability in co-solvent systems containing hydrophobic NADESs. The NADES-tetrahydrofuran (50%, v/v) mixture maintained a high conversion rate (97.32–99.00%) and excellent 6′-regioselectivity (>99%) under optimal conditions (20 mg·mL^−1^ whole-cell dosage, 15:1 vinyl cinnamate/phillyrin, at 50 °C). Crucially, *Aspergillus oryzae* whole-cell biocatalysts exhibited markedly enhanced operational stability across all tested NADES-tetrahydrofuran (50%, v/v) reaction systems, retaining 80.09–97.95% of their original activity after six consecutive batch cycles. This study successfully combined hydrophobic NADESs with a whole-cell biocatalytic system, achieving efficient and regioselective cinnamoylation of phillyrin.

## 1 Introduction

Phillyrin (forsythin), a major bioactive constituent of *Forsythiae Fructus*, exhibits a broad spectrum of pharmacological activities, including antiviral, antibacterial, and anti-inflammatory properties (Wang et al., 2018; Zhang et al., 2018; Ma et al., 2020; Qin et al., 2023; Zhang et al., 2024; Wei et al., 2025). Despite its considerable therapeutic potential, the clinical application of phillyrin is significantly limited by its poor aqueous solubility, low membrane permeability, and susceptibility to P-glycoprotein-mediated efflux, all of which contribute to its low oral bioavailability (Li et al., 2011). Selective acylation of natural polyhydroxylated compounds has emerged as an effective strategy for modulating lipophilicity, thereby improving physicochemical properties and consequently enhancing biological activities. Recent studies have demonstrated that the cinnamic acid ester derivative of phillyrin, synthesized via chemical acylation, exhibits significantly greater anti-inflammatory efficacy than the parent compound (Guo et al., 2024).

Compared with conventional chemical synthesis, lipase-catalyzed acylation offers several advantages, including milder reaction conditions, high regioselectivity, and reduced by-product formation (Staudt et al., 2022; Zhang et al., 2022). However, the high production costs associated with isolated enzymes and commercially available lipases greatly limit their industrial application. Consequently, whole-cell biocatalysis has emerged as a robust and cost-effective alternative (Xu et al., 2020; Yang et al., 2020). The intact cellular envelope serves as a natural protective microenvironment that shields intracellular enzymes from solvent-induced denaturation and thereby enhances the operational stability of the biocatalyst (Xu et al., 2020; Zhang et al., 2022). To further optimize non-aqueous biotransformations, solvent engineering based on natural deep eutectic solvents (NADESs) has attracted considerable attention as a green chemistry strategy (Pätzold et al., 2019).

NADESs exemplify the core tenets of green chemistry owing to their distinctive characteristics, including non-volatility, non-toxicity, non-flammability, good biocompatibility, low cost, and ease of preparation (Choi et al., 2011; Thomas and Kayser, 2022; Usmani et al., 2023). Consequently, NADESs have emerged as promising alternatives to conventional industrial solvents in biocatalytic processes (Cao and Su, 2021; Zhou et al., 2021; Prabhune and Dey, 2023). Robust experimental evidence has demonstrated that specific NADESs can simultaneously enhance both catalytic efficiency and structural stability in isolated enzymes and whole-cell biocatalysts (Nian et al., 2020; Peng et al., 2020; Bittner et al., 2022; Kovács et al., 2022). Nevertheless, despite their well-documented sustainability advantages, current biocatalytic applications remain predominantly confined to hydrophilic NADESs, such as urea- or glycerol-based systems. These hydrophilic NADESs are typically employed as solvent systems with precisely controlled water contents, thereby limiting their applicability in the biocatalytic synthesis of glycoside esters. Recently, hydrophobic NADESs have emerged as superior reaction media for lipase-catalyzed ester synthesis (Hollenbach et al., 2020; Cao et al., 2022). *Candida antarctica* lipase B (CALB) has been shown to retain its native secondary structure in hydrophobic NADESs (Cao et al., 2022). Despite these advances, the application of hydrophobic NADESs as co-solvents in whole-cell-catalyzed biosynthesis of complex natural product derivatives remains largely unexplored.

In this study, we designed and prepared seven hydrophobic NADESs using decanoic acid as the hydrogen bond acceptor combined with various hydrogen bond donors, including terpenes and medium-chain fatty alcohols. For the first time, these hydrophobic NADESs were employed as co-solvents in a whole-cell biocatalytic system for the synthesis of pharmacologically valuable phillyrin cinnamoyl esters. We systematically evaluated the physicochemical properties of the NADESs and their effects on catalytic efficiency, regioselectivity, and biocatalyst recyclability. Furthermore, the effects of these hydrophobic solvent systems on cell membrane permeability and surface morphology were investigated to elucidate the underlying mechanisms. This study aims to provide an efficient and sustainable bioprocess strategy for the modification of natural glycosides in non-aqueous media.

## 2 Materials and methods

### 2.1 Biological and chemical materials

Five microbial strains, including *Aspergillus oryzae*, *Rhizopus oryzae*, *Pseudomonas aeruginosa*, *Pseudomonas stutzeri*, and *Pseudomonas fluorescens*, were acquired from the Guangdong Institute of Microbiology located in Guangzhou, China. Phillyrin was sourced from Nantong Feiyu Biotechnology Co., Ltd. located in Nantong, China. Vinyl cinnamate, nerolidol (Ner), geraniol (Ger), and decyl alcohol (Dec) were procured from Tokyo Chemical Industry (Shanghai) Trading Co., Ltd. Thymol (Thy), decanoic acid (DecA), 1-hexanol (Hex), and 1-octanol (Oct) were purchased from Aladdin Co., Ltd. (Shanghai, China). Carvone (Car) was supplied by Shanghai Maclean Biochemical Technology Co., Ltd. (Shanghai, China). All other reagents and chemicals were obtained from commercial sources and were of analytical-grade.

### 2.2 Preparation of whole-cell biocatalysts

The fungal strains *A. oryzae* and *R. oryzae* were pre-cultured on potato dextrose agar (PDA) slants at 28 °C for 72 h. Subsequently, the activated spore suspension was inoculated into 500-mL Erlenmeyer flasks containing 200 mL of fermentation medium composed of 0.6% soybean oil, 0.2% tryptone, 0.5% (NH₄)₂SO₄, and 0.02% CaCl₂. Following a 36 h cultivation period at 30 °C and 180 r∙min^−1^, the fungal cells were harvested by filtration, rinsed twice with sterile distilled water, and subsequently freeze-dried at −50 °C for 24 h. The preparation of the bacterial biocatalysts (*P. aeruginosa*, *P. stutzeri*, *P. fluorescens*) was conducted according to protocols described in our previous study (Yang et al., 2020).

### 2.3 Preparation and physicochemical characterization of NADESs

The hydrophobic NADESs employed in this study (Table 1) were prepared according to a previously published methodology (Cao et al., 2022). In a typical preparation, all components used to prepare NADESs were vacuum-dried at 60 °C for 3 h. The hydrogen-bond acceptor (HBA) and hydrogen-bond donor (HBD) were combined and agitated using a magnetic stirrer at 80 °C and 100 r∙min^−1^, resulting in the formation of a clear, transparent liquid. To ensure anhydrous conditions for subsequent biocatalysis, activated molecular sieves (4 Å) were introduced into the NADESs, which were then stored in hermetically sealed glass vials. The prepared NADESs were characterized by ^1^H NMR spectroscopy (400 MHz) and Fourier-transform infrared (FT-IR) spectroscopy over a wavenumber range of 4,000 cm^−1^-500 cm^−1^. Dynamic viscosity was quantified using a digital rotational viscometer (LVDV-2 T, Shanghai FangRui Instrument Co., Ltd., China) across a precisely controlled temperature gradient of 25 °C-60 °C. The density of the prepared NADESs was measured at 25 °C by a standard pycnometer.

**Table 1:** Synthesis and preparation of NADESs.

Types	NADES	Abbreviation	HBA	HBD	Molar ratio
DecA-Fatty alcohols	Decanoic acid-Hexanol	DecA-Hex	Decanoic acid	1-Hexanol	1:2
	Decanoic acid-Octanol	DecA-Oct	Decanoic acid	1-Octanol	1:2
	Decanoic acid-Decyl alcohol	DecA-Dec	Decanoic acid	Decyl alcohol	1:2
DecA-Terpene	Decanoic acid-Thymol	DecA-Thy	Decanoic acid	Thymol	1:2
	Decanoic acid-Carvone	DecA-Car	Decanoic acid	Carvone	1:2
DecA-Terpenol	Decanoic acid-Nerolidol	DecA-Ner	Decanoic acid	Nerolidol	1:2
	Decanoic acid-Geraniol	DecA-Ger	Decanoic acid	Geraniol	1:2

### 2.4 Procedure for whole-cell mediated cinnamoylation of phillyrin

Biotransformation assays were performed in 2 mL anhydrous reaction systems consisting of phillyrin (15 mmol∙L^−1^), vinyl cinnamate (acyl donor, 45 mmol·L^−1^-375 mmol·L^−1^), the specified solvent matrix, and the lyophilized whole-cell biocatalyst (5 mg·mL^−1^-25 mg·mL^−1^). The reaction mixtures were incubated at 35 °C-55 °C in a thermostatic shaker at 200 r∙min^−1^. Aliquots were periodically withdrawn to monitor the reaction progression. Negative control experiments, performed under identical conditions but omitting the biocatalyst, yielded no detectable esterification products. Conversion (*C*) was defined as the molar ratio of the consumed phillyrin to its initial concentration. The initial reaction rate (V_0_) was calculated from the linear region of the phillyrin depletion curve during the early phase of the biotransformation. Regioselectivity (%) was mathematically determined by dividing the HPLC peak area of the target 6′-O-cinnamoyl phillyrin by the cumulative peak areas of all detected phillyrin esters at a given sampling time. All experimental variants were performed in triplicate.

### 2.5 Operational stability of the whole-cell biocatalyst

The stability of *A. oryzae* whole cells was systematically evaluated through consecutive batch acylation cycles in both pure THF and the NADES-THF (50%, v/v) co-solvent systems. Each initial batch reaction contained 15 mmol∙L^−1^ phillyrin, 225 mmol∙L^−1^ vinyl cinnamate, and 20 mg∙mL^−1^ biocatalyst at 50 °C. Upon completion of a batch cycle, the *A. oryzae* cells were recovered by gentle filtration. To thoroughly eliminate unreacted substrates and residual products, the recovered biomass was meticulously washed with anhydrous THF. The washed biocatalyst was then immediately resuspended in a fresh reaction medium with identically replenished substrate concentrations for the subsequent cycle. The relative activity (%) for each cycle was calculated by normalizing its final conversion against the conversion achieved in the first batch (defined as 100%).

### 2.6 Membrane permeability and SEM analysis of cells incubated in NADES-containing systems

In a typical experiment, the *A. oryzae* whole-cell catalysts (20 mg·mL^−1^) were incubated in THF or NADES-THF (50%, v/v) systems at 200 r·min^−1^ and 50 °C for 24 h. The supernatants were collected from the reaction mixture and the protein concentration was quantified using the BCA Protein Assay Kit (Shanghai Aladdin Bio-Chem Technology Co., Ltd.). This concentration served as an indicator of cell membrane permeability. The harvested cells were lyophilized under vacuum, and characterized by scanning electron microscopy (SEM) using a Hitachi Regulus 8,100 scanning electron microscope (Hitachi High-Tech Corporation, Japan).

### 2.7 Analytical methods

The samples were analyzed by high-performance liquid chromatography (HPLC), with a Shimadzu LC-200C pump with a DAD detector at 277 nm and a Zorbax SB-C18 column (4.6 mm × 250 mm, 5 μm, Agilent, USA). The mobile phase, consisting of acetonitrile and 0.1% formic acid aqueous solution (80/20, v/v) was maintained at a consistent flow rate of 1 mL·min^−1^. Under the specified chromatographic conditions, the retention times for phillyrin and phillyrin 6′-O-cinnamoyl were around 2.339 min and 2.781 min, respectively.

### 2.8 Purification and structural characterization of the target product

**Figure 1:**
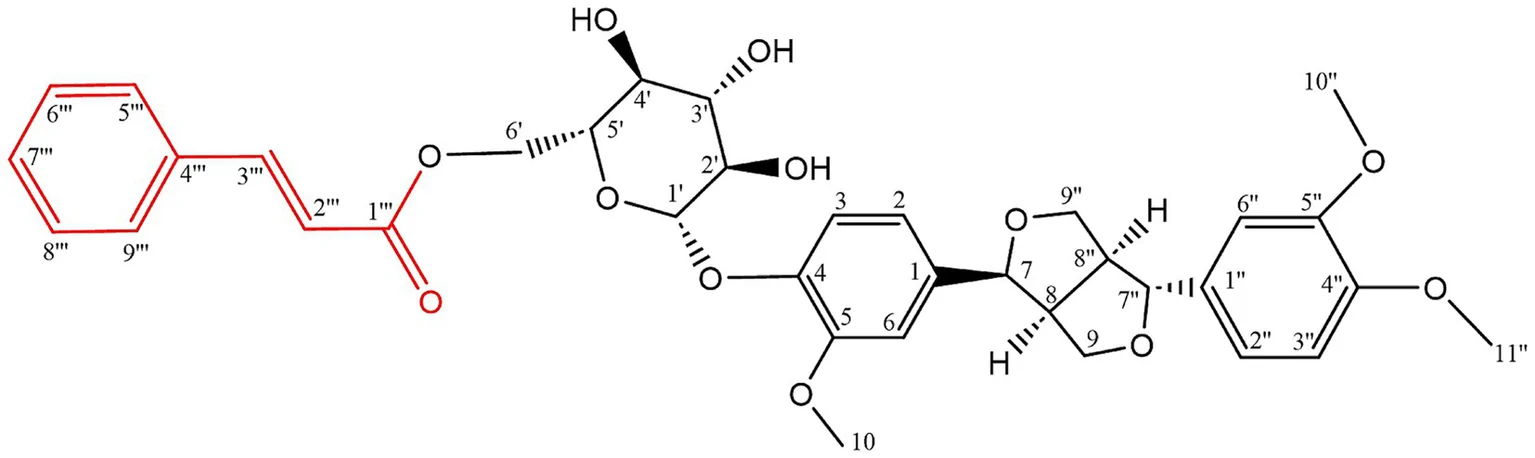
Molecular structure of phillyrin 6’-O-cinnamoyl.

The synthetic product was purified through flash column chromatography using a mixture of ethyl acetate and petroleum ether as the mobile phase. The structure was characterized by ^13^C NMR (100 MHz) and ^1^H NMR (400 MHz) spectroscopy using DMSO-d_6_ as the solvent. The NMR spectroscopic data were provided in the Supplementary Figures S1, S2. The molecular structure of phillyrin 6′-O-cinnamoyl is shown in Figure 1. The product phillyrin 6′-O-cinnamoyl was obtained with an isolated yield of 88%.

^13^C NMR (100 MHz, DMSO): *δ* 166.40 (C_1'''_), 149.22 (C_3''_), 148.96 (C_4''_), 148.13 (C_6_), 146.05 (C_3'''_), 145.01 (C_1_), 135.77 (C_4'''_), 134.51 (C_4_), 131.63 (C_1''_), 130.96 (C_7'''_), 129.43 (C_6'''_+C_8'''_), 128.82 (C_5'''_+C_9'''_), 118.48 (C_3_), 118.38 (C_6''_), 118.06 (C_2'''_), 115.79 (C_2_), 112.06 (C_5''_), 111.17 (C_5_), 109.93 (C_2''_), 100.39 (C_1'_), 87.06 (C_7_), 81.67 (C_7''_), 77.21 (C_3'_), 74.21 (C_5'_), 73.63 (C_2'_), 70.76 (C_4'_), 70.65 (C_9_), 69.28 (C_9''_), 64.09 (C_6'_), 56.14 (C_10_), 55.96 (C_10''_+C_11''_), 54.32 (C_8_), 49.71 (C_8''_). ^1^H NMR (400 MHz, DMSO): *δ* 7.73 (dd, *J* = 6.6, 2.9 Hz, 2H, H_5'''_+H_9'''_), 7.64 (d, *J* = 16.0 Hz, 1H, H_3'''_), 7.50-7.41 (m, 3H, H_6'''_+H_7'''_+H_8'''_), 7.02 (d, *J* = 8.5 Hz, 1H, H_6''_), 6.91 (dd, *J* = 12.9, 5.0 Hz, 3H, H_6_+H_2''_+H_2_), 6.85 (dd, *J* = 8.3, 1.2 Hz, 1H, H_3''_), 6.71 (dd, *J* = 8.4, 1.7 Hz, 1H, H_3_), 6.64 (d, *J* = 16.1 Hz, 1H, H_2'''_), 5.30 (t, *J* = 24.3 Hz, 3H, H_1'_+H_7_+H_7''_), 4.93 (d, *J* = 7.4 Hz, 1H, OH_4'_), 4.71 (d, *J* = 5.9 Hz, 1H, OH_2'_), 4.47-4.39 (m, 1H, OH_3'_), 4.31-4.21 (m, 2H, H_2'_+H_6'_), 3.96 (d, *J* = 9.3 Hz, 1H, H_5'_), 3.80-3.73 (m, 9H, H_10''_+H_11''_+H_9''_+H_9_+H_6'_), 3.72-3.58 (m, 3H, H_10_), 3.34 (d, *J* = 6.4 Hz, 2H, H_9_+H_9''_), 3.28-3.21 (m, 1H, H_3'_), 3.20-3.10 (m, 1H, H_4'_), 3.01 (t, *J* = 8.5 Hz, 1H, H_8_), 2.61 (dd, *J* = 15.5, 6.4 Hz, 1H, H_8''_).

## 3 Results and discussion

### 3.1 Characterization of NADESs

The physicochemical characterization was performed on the seven hydrophobic NADESs. All systems remained homogeneous, stable liquids at room temperature (25 °C). ^1^H NMR and FT-IR were employed to elucidate the strength of intermolecular interactions in NADESs.

#### 3.1.1 Thermophysical properties

**Figure 2:**
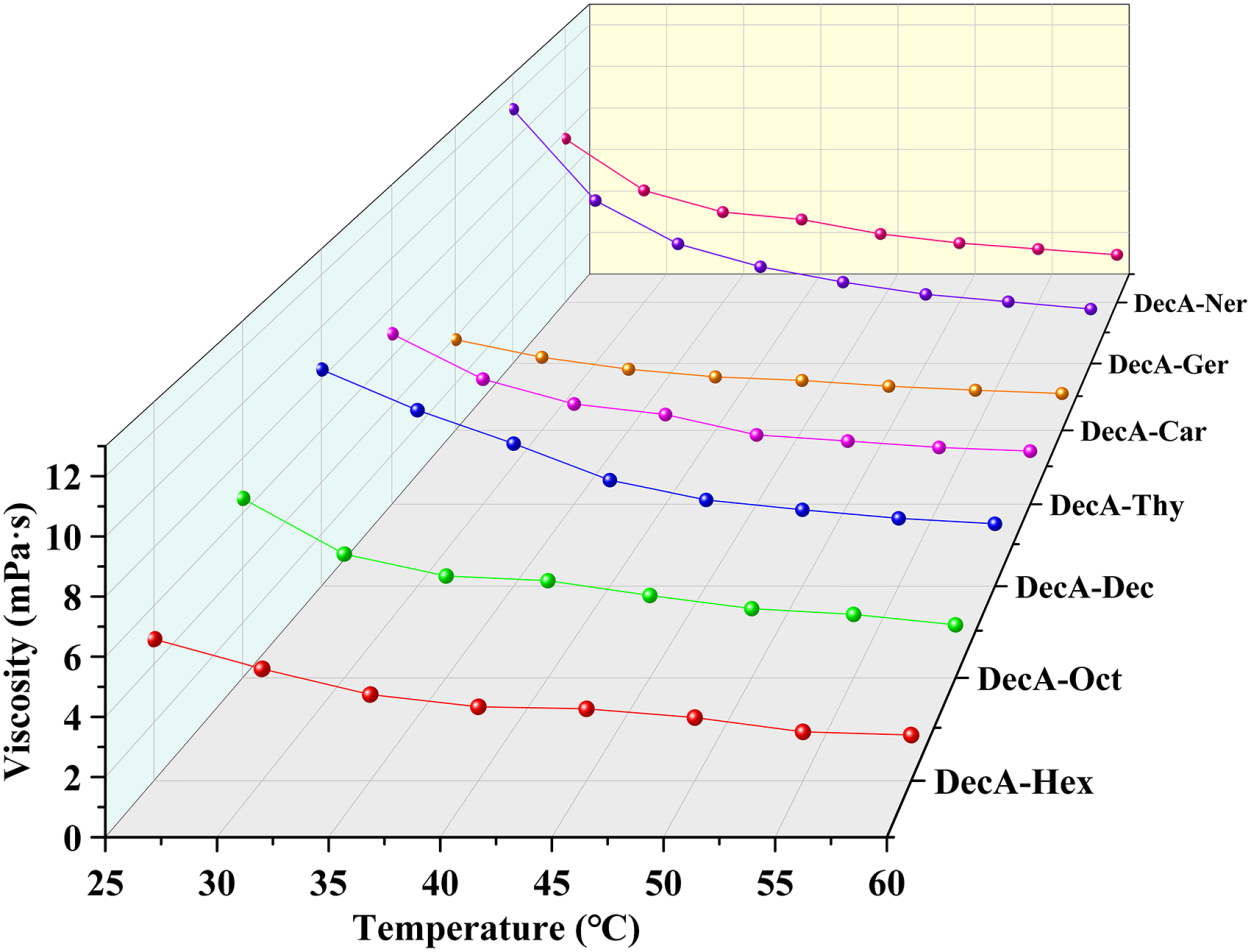
The viscosity-temperature relationship curve of NADESs.

Thermophysical properties, such as viscosity and density, are critical characteristics of any solvent because they influence mass transport phenomena and thereby determine the suitability of solvents for specific applications. The densities of the seven hydrophobic NADESs range from 0.852 to 0.958 g·mL^−1^ (Supplementary Table S1), all lower than that of water (1.000 g·mL^−1^ at 25 °C). The temperature-dependent viscosity of the prepared hydrophobic NADESs was experimentally determined (Figure 2), revealing a consistent decrease in viscosity with increasing temperature. This behavior is consistent with the well-established principle that elevated temperatures enhance molecular mobility and weaken intermolecular interactions, thereby reducing viscosity. As shown in Figure 2, their viscosities range from 1.57 to 11.42 mPa·s across the 25 °C-60 °C interval, substantially lower than those reported for conventional hydrophilic DESs (Huang et al., 2021; Cao et al., 2022). This low-viscosity profile promotes efficient mass transfer and uniform mixing, thereby enhancing their utility as environmentally benign reaction media.

Some studies have suggested that the molecular weight and size of the components can significantly influence the mobility of the entire system, thereby increasing its viscosity (Zhang et al., 2012; Ijardar, 2020). In this case, nerolidol has a higher molecular weight than geraniol, thymol, and carvone (MW_Ner_ = 222.37 g·mol^−1^, MW_Ger_ = 154.25 g·mol^−1^, MW_Thy_ = 150.22 g·mol^−1^, MW_Car_ = 150.22 g·mol^−1^), contributing to the high viscosity of DecA-Ner. For instance, the viscosity of DecA-Ner (11.42 mPa·s) at 25 °C is significantly higher than that of DecA-Ger (7.71 mPa·s), DecA-Thy (6.94 mPa·s), and DecA-Car (3.89 mPa·s). Similarly, the viscosity of DecA-fatty alcohol-type NADESs increases linearly with the elongation of the fatty alcohol alkyl chain, which is attributable to the concomitant increase in molecular weight and size (Florindo et al., 2018).

#### 3.1.2 ^1^H NMR and FT-IR spectra

The ^1^H NMR spectra of the NADESs and their precursor components were detailed in the Supplementary Figures S3–S17. The results showed that all ^1^H NMR resonances in the prepared NADESs were fully assignable to the starting components, with no new signals detected, indicating that NADES formation is driven by intermolecular interactions rather than chemical reactions. This conclusion is supported by robust literature evidence indicating that NADESs are formed via directional hydrogen-bonding networks between their molecular precursors (Zhang et al., 2012; Duque et al., 2023).

Moreover, the formation of NADESs can be confirmed by characteristic shifts and broadening of bands associated with hydrogen-bonding interactions between the HBA and HBD. Supplementary Figures S18–S24 present the FT-IR spectra of the NADESs and their respective components. As exemplified by the DecA-Thy NADES, the O-H stretching band exhibits pronounced broadening and a distinct blue shift (from 3243.68 cm^−1^-3480.88 cm^−1^) relative to that of thymol; concurrently, the C=O stretching band undergoes a clear blue shift from 1698.98 cm^−1^-1708.62 cm^−1^. The FT-IR spectral changes provide compelling evidence for strong intermolecular hydrogen bonding between decanoic acid and the respective hydrogen-bond donors. These findings are in good agreement with previous spectroscopic studies on carboxylic acid-alcohol-based NADESs (Zhang et al., 2018; Li et al., 2024).

### 3.2 Whole-cell catalyzed cinnamoylation of phillyrin in organic solvents

Initially, several lipase-producing microbial strains, commonly utilized in the food, pharmaceutical, and cosmetic industries, were evaluated for their catalytic efficiency in the regioselective cinnamoylation of phillyrin (Table 2). The experimental results demonstrated that whole-cell catalysts derived from five tested strains displayed phillyrin cinnamoylation activity, although substantial differences in catalytic efficiency were evident across the strains. For instance, *A. oryzae* exhibited the highest catalytic efficiency, with an initial reaction rate of 5.68 mmol·L^**−**1^·h^**−**1^ and a substrate conversion rate reaching 95.54%. *P. aeruginosa* and *P. stutzeri* followed in order of performance, displaying initial reaction rates of 3.19 mmol·L^−1^·h^−1^ and 2.46 mmol·L^**−**1^·h^**−**1^, respectively, along with conversion rates of 89.57 and 70.83%. In contrast, *R. oryzae* and *P. fluorescens* demonstrated comparatively lower catalytic efficiencies, which may be attributed to differences in substrate specificity among the cell-bound enzymes derived from distinct microbial origins. Moreover, when vinyl esters serve as acyl donors, the concomitant release of acetaldehyde, a well-established inactivating agent, may induce varying degrees of enzyme deactivation depending on the microbial source. Notably, absolute regioselectivity was consistently observed at the 6′-hydroxyl group of the glucose moiety of phillyrin across all whole-cell catalysts. Consistent with these observations, our recent study also demonstrated that *A. oryzae* whole-cell biocatalysts exhibit high regioselectivity toward the primary hydroxyl group located on the glucose moiety of salidroside (Yang et al., 2022).

**Table 2:** Regioselective cinnamoylation of phillyrin catalyzed by whole cells.

Strains	V_0_ (mmol·L^−1^·h^−1^)	*C* (%)	6’-Regioselectivity (%)
*Aspergillus oryzae*	5.68 ± 0.06	95.54 ± 0.68	>99
*Rhizopus oryzae*	0.86 ± 0.04	17.28 ± 0.96	>99
*Pseudomonas aeruginosa*	3.19 ± 0.08	89.57 ± 1.08	>99
*Pseudomonas stutzeri*	2.46 ± 0.03	70.83 ± 0.57	>99
*Pseudomonas fluorescens*	0.97 ± 0.10	19.24 ± 1.13	>99

Reaction conditions: 15 mmol·L^−1^ phillyrin, 180 mmol·L^−1^ vinyl cinnamate, 20 mg·mL^−1^ catalyst preparation, 2 mL anhydrous acetonitrile, 45 °C, 200 r·min^−1^.

To further elucidate the catalytic performance of the *A. oryzae* whole-cell biocatalyst, the effects of various critical reaction conditions on the cinnamoylation of phillyrin were comprehensively examined. Organic solvents play a crucial role in non-aqueous catalytic processes, significantly influencing the activity, selectivity, and stability of the biocatalysts. Six conventional organic solvents with varying logP values were selected to investigate the effect of solvent polarity on phillyrin cinnamoylation catalyzed by *A. oryzae* whole-cell biocatalysts (Table 3). No catalytic activity was observed in the highly polar solvents dimethyl sulfoxide (DMSO) and N, N-dimethylformamide (DMF), as their high polarity can readily disrupt the essential hydration layer surrounding the enzymes in *A. oryzae* whole cells, leading to inactivation of the whole-cell biocatalysts. This result is consistent with established literature on solvent-induced inactivation of whole-cell biocatalysts (Yang et al., 2020; Yang et al., 2022). *A. oryzae* whole-cell biocatalysts demonstrated superior catalytic performance across all four remaining organic solvent systems, with conversion rates ranging from 89.55 to 98.26%. Tetrahydrofuran (THF) supported the highest catalytic efficiency, achieving a substrate conversion of 98.26% and an initial reaction rate of 6.05 mmol·L^**−**1^·h^**−**1^. Thus, organic solvents may exert differential adverse effects on cell membranes, thereby compromising the structural integrity of intracellular enzymes and leading to their partial or complete inactivation during biocatalytic transformations.

**Table 3:** Effects of organic solvents on cinnamoylation of phillyrin catalyzed by *A. oryzae* whole-cell biocatalysts.

Solvents	logP	V_0_ (mmol·L^−1^·h^−1^)	*C* (%)	6’-Regioselectivity (%)
Acetone	−0.23	5.36 ± 0.04	92.82 ± 0.43	99
Acetonitrile	−0.30	5.68 ± 0.06	95.54 ± 0.68	99
*tert*-Butanol	0.70	4.23 ± 0.11	89.55 ± 1.26	99
Tetrahydrofuran	0.49	6.05 ± 0.09	98.26 ± 0.55	99
Dimethyl sulfoxide	−1.30	n.d.	n.d.	n.d.
N, N-Dimethylformamide	−1.00	n.d.	n.d.	n.d.

Reaction conditions: 15 mmol·L^−1^ phillyrin, 180 mmol·L^−1^ vinyl cinnamate, 20 mg·mL^−1^ *A. oryzae* whole cells, 2 mL anhydrous solvent, 45 °C, 200 r·min^−1^; n.d.: not detected.

**Figure 3:**
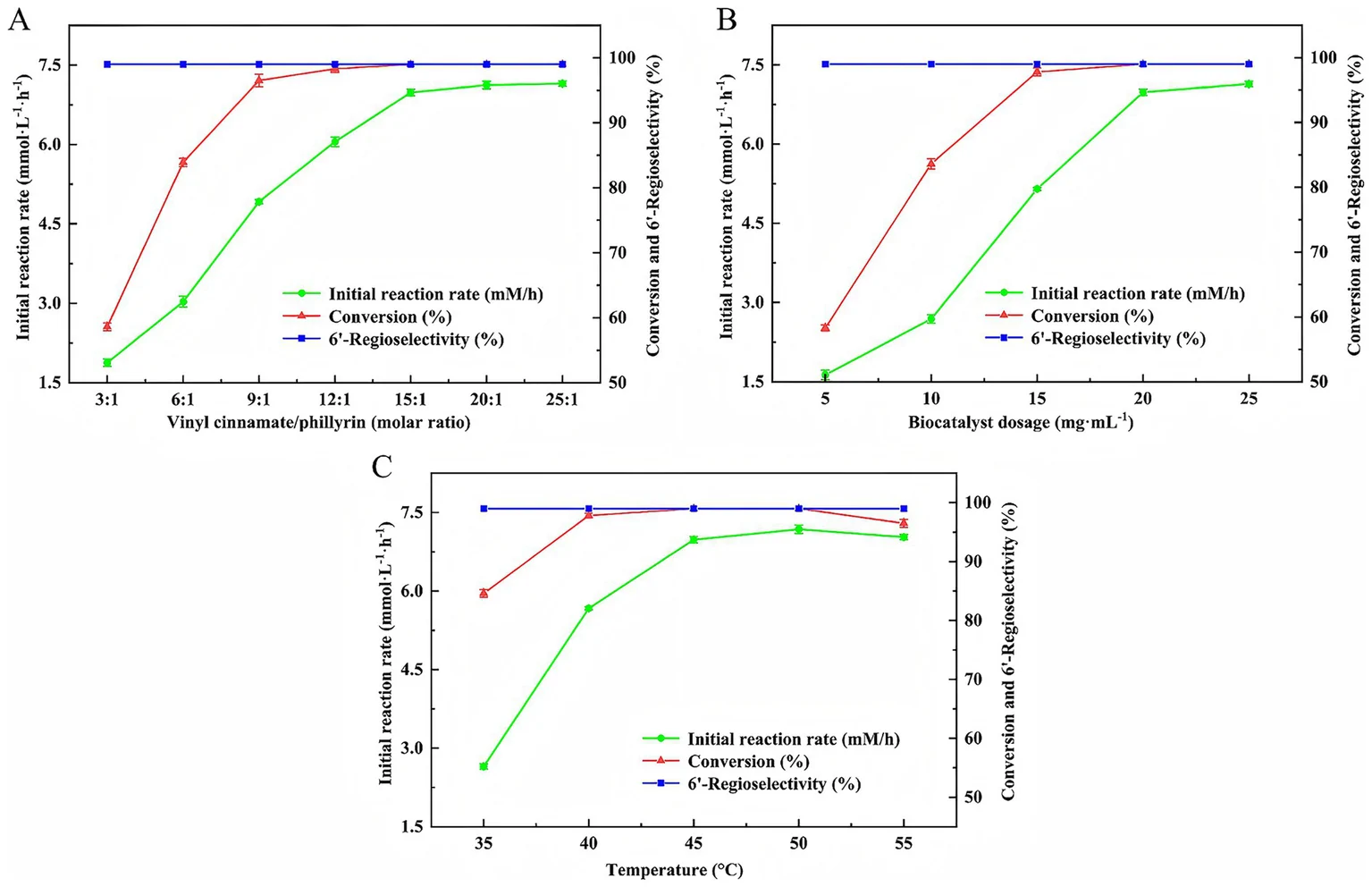
Effect of several key reaction parameters on the cinnamoylation of phillyrin catalyzed by *A. oryzae* whole cells. **(A)** Molar ratio of substrate: The reaction was conducted in anhydrous THF (2 mL) containing phillyrin (15 mmol·L^−1^), vinyl cinnamate (45 mmol·L^−1^–375 mmol·L^−1^) and whole-cell dosage (20 mg·mL^−1^) at 45 °C with shaking at 200 r·min^−1^. **(B)** Dosage of whole-cell biocatalyst: The reaction was conducted in anhydrous THF (2 mL) with phillyrin (15 mmol·L^−1^), vinyl cinnamate (225 mmol·L^−1^) and whole-cell catalysts (5 mg·mL^−1^–25 mg·mL^−1^) at 45 °C with shaking at 200 r·min^−1^. **(C)** Reaction temperature: The reaction was conducted in anhydrous THF (2 mL) with phillyrin (15 mmol·L^−1^), vinyl cinnamate (225 mmol·L^−1^) and whole-cell catalysts (20 mg·mL^−1^) under varying temperatures (35 °C–55 °C) with shaking at 200 r·min^−1^.

As shown in Figure 3A, the molar ratio of vinyl cinnamate to phillyrin significantly influenced both the initial acylation rate and the maximum conversion of phillyrin. Increasing the vinyl cinnamate-to-phillyrin ratio enhanced both the conversion yield and the initial acylation rate. For example, increasing the molar ratio of vinyl cinnamate to phillyrin from 3:1 to 15:1 increased the initial acylation rate from 1.88 to 6.98 mmol·L^**−**1^·h^**−**1^ and raised the conversion yield from 58.60 to 99%. Given that the cinnamoylation of phillyrin is a bimolecular reaction, an excess of vinyl cinnamate significantly enhances reaction efficiency by driving the equilibrium toward product formation. Moreover, as enzymatic hydrolysis of vinyl cinnamate constituted a competing side reaction, the acyl donor was supplied in excess of the stoichiometric amount. As demonstrated in Figure 3B, whole-cell dosage significantly influenced both the initial acylation rate and the conversion yield, with a more pronounced effect on the initial acylation rate. Across the tested dosage range of 5 to 20 mg·mL^**−**1^, the initial acylation rate increased linearly with increasing whole-cell dosage, primarily due to the high substrate concentration during the early reaction phase. At a whole-cell dosage of 20 mg·mL^**−**1^, the initial acylation rate reached 6.98 mmol·L^**−**1^·h^**−**1^, with a conversion yield of 99%. Temperature generally influences both the enzymatic activity and the conformational stability of biocatalysts. As illustrated in Figure 3C, the initial acylation rate increased sharply over the temperature range of 35 °C-50 °C, from 2.65 to 7.18 mmol·L^**−**1^·h^**−**1^. Notably, both the conversion yield and the initial acylation rate decreased markedly above 50 °C. This suggests that elevated reaction temperatures enhance mass transfer between the reaction medium and the cellular membranes and promote substrate diffusion to the active sites. However, excessive temperatures may induce conformational changes in the enzymes, leading to partial inactivation. Based on the experimental results, the optimal conditions were determined as a whole-cell dosage of 20 mg·mL^**−**1^, a vinyl cinnamate-to-phillyrin molar ratio of 15, and a reaction temperature of 50 °C. Notably, high regioselectivity toward the 6’-OH position of phillyrin was consistently observed across all tested conditions.

### 3.3 Whole-cell catalyzed phillyrin cinnamoylation in NADES systems

Cosolvent systems, including ionic liquid-organic solvent combinations, are increasingly recognized as an effective strategy for the enzymatic synthesis of glucoside ester derivatives (Yang et al., 2020). Given the higher viscosity of NADESs and the limited solubility of phillyrin in hydrophobic NADESs, NADES-THF co-solvents were employed as the reaction medium (Table 4). Notably, in NADES-THF co-solvent systems containing DecA-fatty alcohols, an optimal NADES concentration (30%, v/v) yielded the highest initial reaction rates. In the DecA-Oct and DecA-Dec systems, the initial reaction rate exceeded those observed in the DecA-Hex system. Consistent with prior reports, the initial rate of CALB-catalyzed transesterification in decanoic acid-fatty alcohol NADES increased progressively with increasing alkyl chain length of the fatty alcohol (Cao et al., 2022). In NADES systems based on DecA-Terpene or DecA-Terpenol, the maximum initial reaction rate was achieved at 10% (v/v). Generally, reaction rates were influenced by the viscosity of the reaction medium. The hydrophobic NADESs exhibited viscosities of 1.89 mPa·s-3.10 mPa·s at 50 °C, markedly higher than that of THF under identical conditions, indicating that viscosity is not the sole factor determining reaction rates. Instead, the differences in reaction efficiency likely stem from synergistic effects involving enhanced cell membrane permeability and solvent-mediated modulation of the enzyme microenvironment. Remarkably, *A. oryzae* whole cells demonstrated high catalytic activity across all investigated NADES-THF medium systems. Even when the NADES concentration was increased to 50% (v/v), substrate conversion consistently remained above 97%, and strict 6′-regioselectivity (>99%) was maintained. Previous studies have demonstrated that hydrophobic NADESs are more effective media than hydrophilic NADESs for lipase-catalyzed ester synthesis (Hollenbach et al., 2020; Cao et al., 2022). For instance, Hollenbach et al. (2020) demonstrated that hydrophobic DESs exhibited superior performance as reaction media compared with hydrophilic DESs in CALB-catalyzed glycolipid synthesis. Specifically, the yield of glucose monodecanoate in the hydrophobic DES system composed of menthol and decanoic acid was 20–1,000 times higher than that obtained in hydrophilic DESs. Moreover, hydrophobic DESs induced less inactivation of lipase CALB compared with hydrophilic DESs, and the highest yield of benzyl acetate in hydrophobic DESs reached 56.75% after 12 h (Cao et al., 2022). This difference may result in a pronounced reduction in enzyme activity in hydrophilic NADESs relative to hydrophobic NADESs, as highly polar solvents tend to disrupt the essential hydration shell surrounding the enzyme (Oh et al., 2019; Hollenbach et al., 2020).

**Table 4:** Cinnamoylation of phillyrin by *A. oryzae* whole cells in THF-NADES systems.

Solvents	V_0_ (mmol·L^−1^·h^−1^)	*C* (%)	6’-Regioselectivity (%)
THF	7.18 ± 0.08	99	>99
THF-[DecA-Hex] (90:10)	7.42 ± 0.11	99	>99
THF-[DecA-Hex] (70:30)	7.49 ± 0.13	99	>99
THF-[DecA-Hex] (50:50)	7.15 ± 0.17	98.64 ± 0.26	>99
THF-[DecA-Oct] (90:10)	7.45 ± 0.09	99	>99
THF-[DecA-Oct] (70:30)	8.40 ± 0.14	99	>99
THF-[DecA-Oct] (50:50)	8.22 ± 0.17	99	>99
THF-[DecA-Dec] (90:10)	8.26 ± 0.11	99	>99
THF-[DecA-Dec] (70:30)	8.39 ± 0.15	99	>99
THF-[DecA-Dec] (50:50)	8.24 ± 0.19	99	>99
THF-[DecA-Thy] (90:10)	8.13 ± 0.12	99	>99
THF-[DecA-Thy] (70:30)	7.93 ± 0.16	99	>99
THF-[DecA-Thy] (50:50)	7.11 ± 0.19	99	>99
THF-[DecA-Car] (90:10)	7.73 ± 0.11	99	>99
THF-[DecA-Car] (70:30)	7.26 ± 0.18	99	>99
THF-[DecA-Car] (50:50)	7.08 ± 0.22	98.53 ± 0.33	>99
THF-[DecA-Ner] (90:10)	7.97 ± 0.12	99	>99
THF-[DecA-Ner] (70:30)	7.86 ± 0.17	99	>99
THF-[DecA-Ner] (50:50)	7.09 ± 0.23	99	>99
THF-[DecA-Ger] (90:10)	8.04 ± 0.13	99	>99
THF-[DecA-Ger] (70:30)	7.93 ± 0.16	98.37 ± 0.31	>99
THF-[DecA-Ger] (50:50)	7.27 ± 0.18	97.32 ± 0.42	>99

Reaction conditions: 15 mmol·L^−1^ phillyrin, 225 mmol·L^−1^ vinyl cinnamate, 20 mg·mL^−1^ *A. oryzae* whole cells, 2 mL THF-NADES, 50 °C, 200 r·min^−1^.

### 3.4 Operational stability of whole-cell biocatalysts in NADES-containing systems

**Figure 4:**
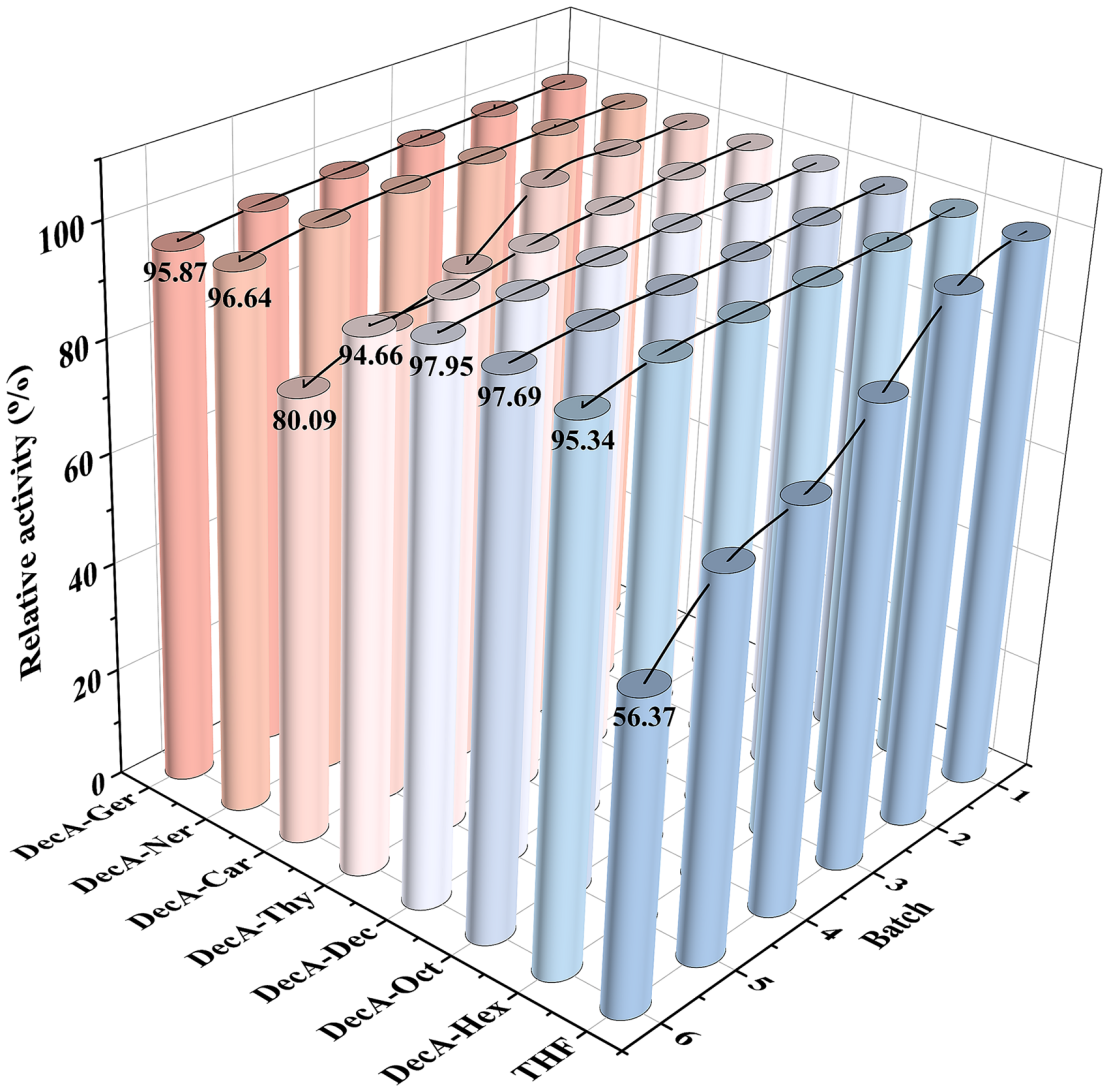
The operational stability of *A. oryzae* whole cells in THF-NADES or THF systems.

From a process engineering perspective, robust operational stability and reusability are critical economic factors for the industrial implementation of whole-cell biocatalysts. Consequently, the recycling performance of *A. oryzae* whole cells was evaluated in the NADES-THF (50%, v/v) co-solvent systems over successive batch cycles. As shown in Figure 4, the incorporation of hydrophobic NADESs significantly improved biocatalyst stability compared to the pure THF system. In pure THF, the residual catalytic activity decreased to 56.37% after six consecutive cycles. In all NADES-THF (50%, v/v) systems (except for DecA-Car), the biocatalyst retained over 94% of its initial activity under identical operating conditions. This enhanced stability may be attributed to the disruption of the essential hydration shell surrounding intracellular enzymes by polar organic solvents (such as THF), which can lead to irreversible alterations in their three-dimensional conformation. The hydrophobic NADESs likely modulate cell envelope permeability and provide a protective microenvironment, restricting excessive THF diffusion and thereby mitigating solvent-induced dehydration. Previous studies have also reported that the lipase iCalB exhibits high operational stability in the hydrophobic NADES menthol/decanoic acid (1:2), retaining full activity over at least five consecutive reaction cycles (Idris and Bukhari, 2012; Hollenbach et al., 2020). Collectively, these results further support the application potential of hydrophobic NADESs as green and sustainable reaction media for industrial biocatalysis.

### 3.5 Effect of NADES-containing systems on protein leakage and surface morphology of *A. oryzae* whole cells

**Figure 5:**
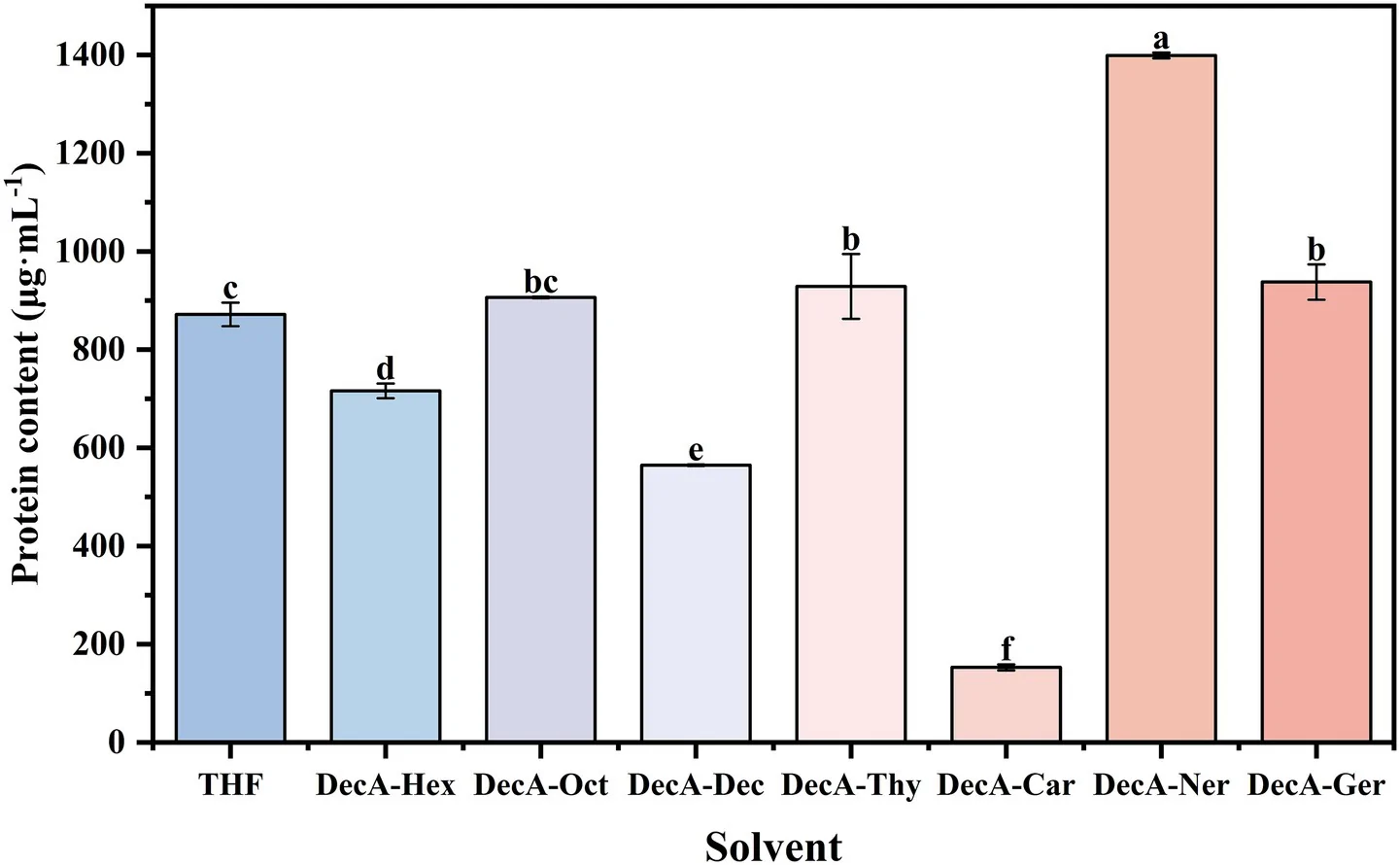
Effect of NADESs on protein leakage from *A. oryzae* whole cells in THF-NADES systems. Different letters showed significant differences (*p* < 0.05) in multiple-range analysis. The experiments were carried out in triplicate, and the data are expressed as the mean ± SD.

**Figure 6:**
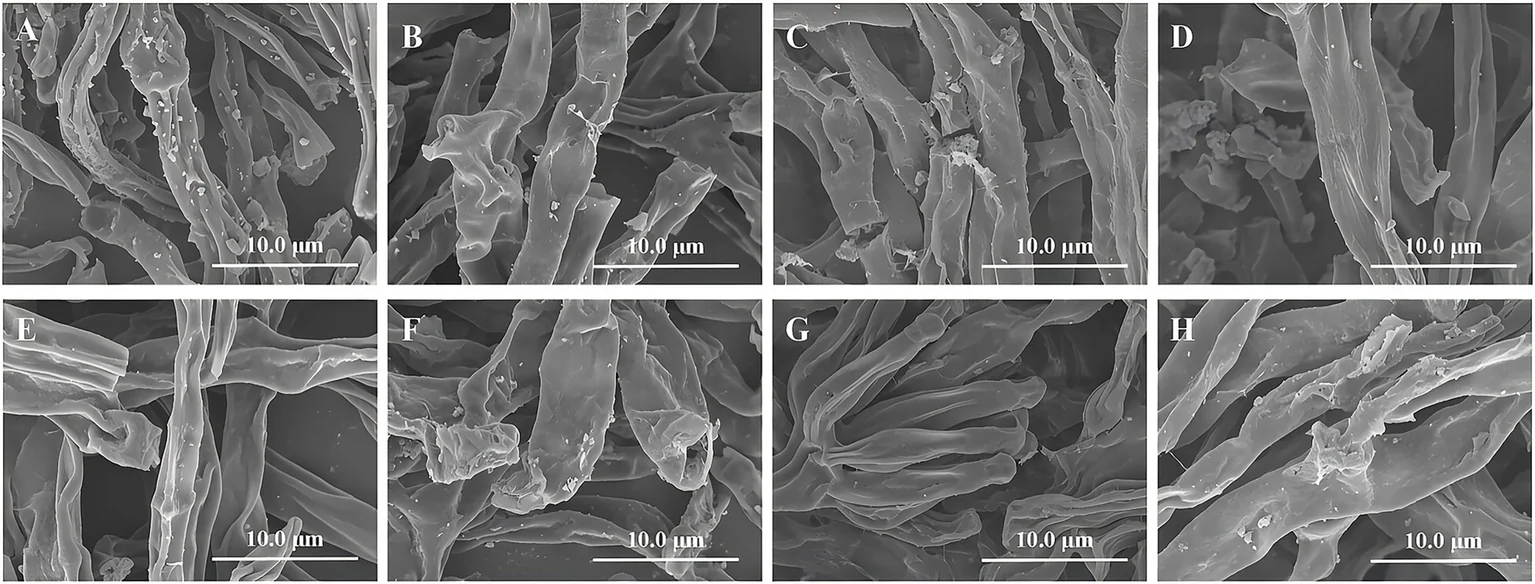
Effect of NADESs on cellular morphology in THF-NADES systems. **(A)**: THF; **(B)**: DecA-Hex; **(C)**: DecA-Oct; **(D)**: DecA-Dec; **(E)**: DecA-Thy; **(F)**: DecA-Car; **(G)**: DecA-Ner; **(H)**: DecA-Ger.

To elucidate the mechanistic basis underlying the altered catalytic performance in NADES-containing systems, cell envelope integrity was evaluated by quantifying extracellular protein leakage. The addition of specific NADESs significantly affected membrane permeability (Figure 5). Compared with pure THF, which induced a protein leakage level of 871.79 μg·mL^−1^, DecA-Ner significantly increased protein leakage to 1399.05 μg·mL^−1^, whereas DecA-Car markedly reduced it to 152.79 μg·mL^−1^. Similar observations have been reported, indicating that specific hydrophobic NADESs disrupt cell membrane integrity, leading to a significant increase in membrane permeability (Cao et al., 2020). Morphological characterization by SEM further supported these findings (Figure 6). Exposure to pure THF caused severe cellular deformation and pronounced surface wrinkling in *A. oryzae* cells (Figure 6A). In contrast, the incorporation of NADESs into the THF matrix preserved cellular morphology and alleviated solvent-induced surface wrinkling (Figures 6B–H). These findings demonstrate that specific hydrophobic NADESs not only enhance substrate and product mass transfer by modulating cell membrane permeability but also maintain cellular structural integrity, thereby protecting intracellular enzymes from inactivation caused by harsh organic solvents. Previous studies have shown that menthol-oleic acid suspensions selectively perturb the *Escherichia coli* cell membrane, inducing localized defects in the phospholipid bilayer without causing complete membrane disintegration, thereby allowing partial leakage of intracellular components (Cao et al., 2020).

## 4 Conclusion

In this study, efficient cinnamoylation of phillyrin was achieved using *A. oryzae* whole cells in NADES-THF co-solvent systems. Crucially, the incorporation of hydrophobic NADESs effectively mitigated solvent-induced biocatalyst deactivation, preserving 80.09–97.95% of the initial catalytic activity after six consecutive reaction cycles, a level that was substantially higher than the residual activity observed in pure THF (56.37%). The hydrophobic NADESs not only enhanced substrate and product mass transfer by modulating cell membrane permeability, but also maintained cellular structural integrity, thereby protecting intracellular enzymes from denaturation induced by strong organic solvents. These findings provide valuable insights into the structural modification of high-value pharmacologically active glycosides in NADESs.

## Data Availability

The original contributions presented in the study are included in the article/Supplementary material, further inquiries can be directed to the corresponding author.
