# Supplementary material for: Whole-cell-catalyzed cinnamoylation of phillyrin in natural deep eutectic solvent-based co-solvent systems
Source: Front Microbiol. 2026 Aug 21;17:1906247. doi: 10.3389/fmicb.2026.1906247 (PMC13541702; doi:10.3389/fmicb.2026.1906247)
Supplement: Supplementary file 1 [file Data_Sheet_1.docx]

Supplementary Material

# 1 **Dehydration of the reaction medium**

The activated molecular sieves were employed to establish a nearly anhydrous reaction medium. The dehydration procedure was carried out as follows: 4 Å molecular sieves were activated at 350 ℃ for 4 h, then cooled to room temperature in a desiccator. Subsequently, the reaction solvent was added at a mass ratio of 50% (w/w) relative to the molecular sieves. The mixture was shaken at 70 r·min^-1^ for 24 h at room temperature, and the molecular sieves were separated to afford the dehydrated reaction medium.

# 2 Supplementary Figures and Tables

## 2.1 Supplementary Table

**Supplementary Table 1.** Densities of the NADESs.

| Types | Abbreviation | Molar ratio | Density(g·mL^-1^) |
| --- | --- | --- | --- |
| DecA-fatty alcohols | DecA-Hex | 1:2 | 0.852 ± 0.005 |
|  | DecA-Oct | 1:2 | 0.853 ± 0.007 |
|  | DecA-Dec | 1:2 | 0.854 ± 0.004 |
| DecA-Terpene | DecA-Thy | 1:2 | 0.958 ± 0.013 |
|  | DecA-Car | 1:2 | 0.944 ± 0.012 |
| DecA-Terpenol | DecA-Ner | 1:2 | 0.894 ± 0.009 |
|  | DecA-Ger | 1:2 | 0.902 ± 0.010 |

## 2.2 Supplementary Figures

**Supplementary Figure 1.** ^1^H NMR spectra of phillyrin 6'-O-cinnamoyl in DMSO-d_6_.

**Supplementary Figure 2.** ^13^C NMR spectra of NMR spectra of phillyrin 6'-O-cinnamoyl in DMSO-d_6_.

**Supplementary Figure 3.** ^1^H NMR spectra of Decanoic acid.

**Supplementary Figure 4.** ^1^H NMR spectra of 1-Hexanol.

**Supplementary Figure 5.** ^1^H NMR spectra of Decanoic acid:1-Hexanol 1:2.

**Supplementary Figure 6.** ^1^H NMR spectra of 1-Octanol.

**Supplementary Figure 7.** ^1^H NMR spectra of Decanoic acid:1-Octanol 1:2.

**Supplementary Figure 8.** ^1^H NMR spectra of Decyl alcohol.

**Supplementary Figure 9.** ^1^H NMR spectra of Decanoic acid:Decyl alcohol 1:2.

**Supplementary Figure 10.** ^1^H NMR spectra of Thymol.

**Supplementary Figure 11.** ^1^H NMR spectra of Decanoic acid:Thymol 1:2.

**Supplementary Figure 12.** ^1^H NMR spectra of Carvone.

**Supplementary Figure 13.** ^1^H NMR spectra of Decanoic acid:Carvonel 1:2.

**Supplementary Figure 14.** ^1^H NMR spectra of Nerolidol.

**Supplementary Figure 15.** ^1^H NMR spectra of Decanoic acid:Nerolidol 1:2.

**Supplementary Figure 16.** ^1^H NMR spectra of Geraniol.

**Supplementary Figure 17.** ^1^H NMR spectra of Decanoic acid:Geraniol 1:2.

**Supplementary Figure 18.** Fourier transform infrared (FT-IR) spectra of Decanoic acid+1-Hexanol.

**Supplementary Figure 19.** Fourier transform infrared (FT-IR) spectra of Decanoic acid+1-Octanol.

**Supplementary Figure 20.** Fourier transform infrared (FT-IR) spectra of Decanoic acid+Decyl alcohol.

**Supplementary Figure 21.** Fourier transform infrared (FT-IR) spectra of Decanoic acid+Thymol.

**Figure S22.** Fourier transform infrared (FT-IR) spectra of Decanoic acid+Carvonel.

**Figure S23.** Fourier transform infrared (FT-IR) spectra of Decanoic acid+Nerolidol.

**Figure S24.** Fourier transform infrared (FT-IR) spectra of Decanoic acid+Geraniol.
